# Anxiety and depression in patients aged 80 years and older following aortic valve therapy. A six-month follow-up study

**DOI:** 10.1007/s40520-023-02541-5

**Published:** 2023-08-30

**Authors:** Leslie S. P. Eide, Bengt Fridlund, Karl Ove Hufthammer, Rune Haaverstad, Erik J. S. Packer, Anette H. Ranhoff, David R. Thompson, Tone M. Norekvål, The CARDELIR Investigators

**Affiliations:** 1 Department of Health and Social Sciences, Western Norway University of Applied Sciences, Inndalsveien 28, Post Box 7030, 5020 Bergen, Norway; 2 Centre of Interprofessional Cooperation within Emergency Care (CICE), Linnaeus University, Växjö, Sweden; 3 Department of Heart Disease, Haukeland University Hospital, Bergen, Norway; 4 Centre for Clinical Research, Haukeland University Hospital, Bergen, Norway; 5 Department of Clinical Science, University of Bergen, Bergen, Norway; 6 Kavli Research Center for Geriatrics and Dementia, Haraldsplass Hospital, Bergen, Norway; 7 School of Nursing and Midwifery, Queen’s University Belfast, Belfast, UK

**Keywords:** TAVI, SAVR, Anxiety and depression, HADS, 80 and over

## Abstract

**Background:**

Little is known about mental health following advanced cardiac procedures in the oldest patients.

**Aims:**

To study changes in anxiety and depression from baseline to one- and six-month follow-up in older patients following transcatheter aortic valve implantation (TAVI) or surgical aortic valve replacement (SAVR).

**Methods:**

Prospective cohort study of patients ≥ 80 years undergoing elective TAVI or SAVR in a tertiary university hospital. Anxiety and depression were assessed with the Hospital Anxiety and Depression Scale. Differences between TAVI/SAVR were analyzed using Welch’s *t* test or chi-squared. Changes over time and group differences were established with longitudinal models using generalized least squares.

**Results:**

In 143 patients (83.5 ± 2.7 years), 46% (n = 65) received TAVI. Anxiety was identified in 11% of TAVI patients at baseline. One- and six-months later, percentages were 8% and 9%. In SAVR patients, 18% had baseline scores indicating anxiety. One and six-months later, percentages were 11% and 9%. Depression was identified in 15% of TAVI patients. One- and six-months later, percentages were 11% and 17%. At baseline, 11% of SAVR patients had scores indicating depression. One- and six-months after SAVR, percentages were 15% and 12%. Longitudinal analyses showed reductions (*P* < 0.001) in anxiety from baseline to one-month, and stable scores between one- and six-months for both treatment groups. There was no change over time for depression among treatment groups (*P* = 0.21).

**Discussion and conclusions:**

SAVR or TAVI in patients ≥ 80 years was associated with anxiety reduction between baseline and follow-up. For depression, there was no evidence of change over time in either treatment group.

**Graphical abstract:**

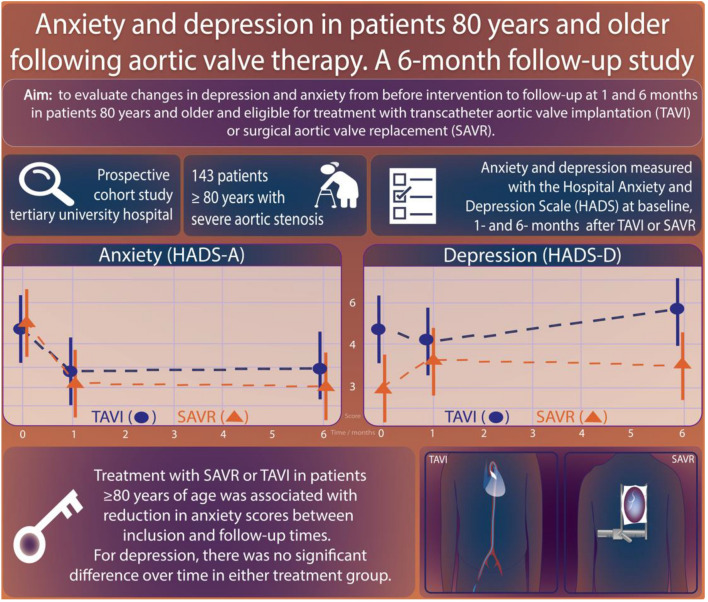

**Supplementary Information:**

The online version contains supplementary material available at 10.1007/s40520-023-02541-5.

## Introduction

Health sciences have traditionally evolved around the concept of single diseases and devoted less effort to understanding the coexistence of somatic and mental health conditions [1, 2]. More than 950 million people worldwide live with mental health disorders such as anxiety and depression [2], conditions that have also been associated with negative physical outcomes for patients in clinical settings [3, 4]. As these conditions are not always identified by healthcare professionals [5], it has been recommended that they be routinely assessed using reliable and valid screening instruments to promote and strengthen their recognition [2, 6]. One such instrument, the Hospital Anxiety and Depression Scale (HADS) [7], provides useful information regarding emotional distress and has been extensively used in clinical studies [8].

Severe aortic stenosis (AS) is a debilitating condition with a prevalence that increases with age [9]. Survival is severely compromised in patients with AS when angina, syncope, and dyspnea appear [10]. Surgical aortic valve replacement (SAVR) is an effective and established treatment of AS but is burdensome in the elderly and in patients with comorbidities, as general anesthesia, sternotomy, and cardiovascular bypass circulation is required. This may explain why SAVR has not been performed in more than 30% of elderly patients with severe symptomatic AS [11]. Transcatheter aortic valve implantation (TAVI) is an option for patients such as the elderly with multi-morbid conditions [12] unable to undergo SAVR. Current European guidelines recommend TAVI in patients more than 75 years of age who are suffering from severe AS [13].

Despite appeals for research into the mental health of older adults [14], limited information exists regarding how anxiety and depression are assessed and reported before and after cardiac invasive intervention [14]. This applies particularly to patients aged 80 years and older due to limited access to effective invasive treatment for AS in the past [15]. The aim of this study was to evaluate changes in anxiety and depression from before the intervention to follow-up at one and six months in patients aged 80 years and older and eligible for SAVR or TAVI.

## Methods

This article presents secondary analyses of data from the larger Delirium in Octogenarians Undergoing Cardiac Surgery or Intervention (CARDELIR) [16] study.

Some results from the CARDELIR study have been reported previously but the aims, outcomes, and results presented in this article are original and are published here for the first time. This article adheres to the STrengthening the Reporting of OBservational studies in Epidemiology (STROBE) guidelines.

The CARDELIR study was a prospective cohort study of individuals undergoing *elective* SAVR or TAVI in a tertiary hospital in western Norway. Follow-up times were one and six months after the procedure was performed. Inclusion criteria were: (1) being aged 80 years or older, (2) willingness to participate in the study, and (3) previous diagnosis of severe aortic stenosis as defined at the time of inclusion [17]. Exclusion criteria were: (1) inability to speak Norwegian and (2) declined consent to participate in the study.

Before hospital admission, a team comprising cardiothoracic surgeons and invasive cardiologists identified individuals unsuitable for SAVR according to guidelines used at the time [17]. Reasons for being considered ineligible for SAVR included comorbidities and risk factors such as previous coronary artery bypass grafting, severe respiratory insufficiency, calcified ascending aorta, prior thoracic radiotherapy, and disabilities reducing the potential for rehabilitation.

From February 2011 until August 2013, 162 individuals aged 80 years or older were treated with SAVR or TAVI. Of these, 15 failed to fulfill the inclusion criteria. The remaining 147 received study information, and 144 agreed to participate. The data set included 143 individuals, as one withdrew consent before treatment. At follow-up, seven were nonresponsive or had died [18].

### Measurements

Anxiety and depression were measured with the Norwegian version of the Hospital Anxiety and Depression Scale (HADS) [7], a valid and reliable instrument [19] extensively used in other studies [19, 20] The HADS consists of 14 items that create two subscales: HADS-Anxiety (HADS-A) and HADS-Depression (HADS-D). The maximum score on each subscale is 21. A score ≥ 8 on the anxiety scale indicates the presence of anxiety while a score ≥ 8 on the depression scale indicates depression [21].

The Barthel Index [22] was used to measure patients’ self-care abilities in ten basic areas: feeding, personal toileting, bathing, dressing, toilet use, bladder and bowel care, ambulation, transfers, and use of stairs. This index is a valid and reliable tool with scores ranging from 0 to 20 [23]. High scores represent higher levels of functioning [22].

The Nottingham Extended Activities of Daily Living Scale [24] is a valid and reliable instrument [25] that assesses patients’ ability to perform tasks requiring higher levels of functioning, such as household management [25]. The scores range from 0 to 66, with low scores representing low levels of independence [24].

We used the Mini-Mental State Examination (MMSE) [26] to assess general cognitive function. The MMSE is a valid and reliable method [27] that uses a scale ranging from 0 to 30. Low scores represent lower levels of functioning [26].

The burden of comorbidity was established with the Charlson Comorbidity Index (CCI) [28]. By assigning a score of 1, 2, 3, or 6 to a set of different comorbidities, the CCI predicts ten-year mortality [28]. The psychometrical properties of the index have been shown in several studies [29].

The Logistic European System for Cardiac Operative Risk Evaluation I (Logistic EuroSCORE I) was used to evaluate cardiac operative risk [30]. This tool uses 17 variables to predict operative mortality. High scores represent a higher risk of mortality [30].

The primary outcome of this study was changes in anxiety or depression in patients aged 80 years and older at one and six months following SAVR or TAVI.

Informed consent was obtained before data collection started and patients were recruited the day before treatment. Preoperative data, including sociodemographic characteristics, ADL and IADL function, MMSE and HADS, were collected the day before treatment with SAVR or TAVI by way of interviews or self-reported forms, as appropriate. Clinical variables were gathered from patient medical records at the time of inclusion.

Follow-up visits were scheduled at the hospital for one and six months after treatment. Information about ADL function was collected at follow-up, and self-report IADL and HADS questionnaires were provided when patients arrived at the hospital. If a patient was unable to attend a follow-up visit and a new appointment could not be scheduled within a two-week window, telephone contact was attempted. Information required for the Barthel Index was collected over the telephone, then the IADL and HADS self-report forms were sent by mail for home completion, together with a pre-stamped and pre-filled envelope for the patient to return the forms to the hospital.

### Statistical analysis

The original sample size calculation was based on a different primary outcome [16]. For the present sub-study, we therefore performed a post-hoc power analysis based the actual sample sizes obtained. We used a simulation-based approach where we simulated normally distributed data with the same variances/covariances as in the original dataset (stratified by HADS domain and treatment group). When there were missing data in the original dataset, data were removed from the simulated data. In the simulations, we assumed that there were no differences from baseline to 30 days, but a mean reduction of 1.7 points at six months for both treatment groups. This value was chosen based on the estimated Minimal Clinically Important Difference (MCID) for HADS in patients with cardiovascular disease [31]. Based on 400 simulations, the power to detect changes over time (using the same omnibus test as in the main analysis) was > 99% for both HADS-A and HADS-D.

Categorical variables are presented as counts and percentages, and continuous variables are presented as means and standard deviations (SD) or confidence intervals. Patients were categorized by treatment group (TAVI/SAVR), and HADS subscale scores are reported both as means and according to a cut-off of 8 or higher (indicating possible anxiety/depression). Differences between groups were analyzed using Welch’s *t* test or chi-squared tests, as appropriate. All confidence intervals are 95% confidence intervals, and tests have not been adjusted for multiple comparisons.

To evaluate changes over time and group differences in anxiety and depression, we used generalized least squares to fit longitudinal models for HADS-A and HADS-D. The models had unstructured residual covariance matrices, and the covariates included were time (categorical, three levels), treatment (TAVI/SAVR), and the time–treatment interaction. To test for changes over time, an omnibus likelihood ratio test comparing the above model with a similar model with only treatment as an explanatory variable was used.

For patients with *partially* missing HADS data (missing items) at a given time point, the missing items were replaced with the means of the answered items in the subscale if at least half of the subscale had been answered, as suggested in the literature [32]. The use of a longitudinal model where we modeled the covariance structure allowed us to also include patients who had reported HADS data at only one or two time points. These models will give unbiased estimates under a “missing at random assumption”, which is much weaker than the “missing completely at random” assumption of complete-case analysis. For each analysis with missing data, we report the number of patients and/or measurements on which the analysis was based.

The longitudinal models were fitted using the “nlme” package version 3.1–161 [33] in R version 4.2.1 [34]. Other analyses were performed using SPSS version 26.0 (IBM SPSS Statistics for Windows, Armonk, NY, USA).

### Ethics

This study was conducted in accordance with the Declaration of Helsinki and approved by the Regional Committee for Ethics in Medical Research in Norway (REK Vest 2010/2936–6).

## Results

Characteristics of included patients (*N* = 143) are shown in Table 1. Fifty-six percent of the participants were female, and TAVI was performed in 46% of patients.

**Table 1:** Baseline characteristics of patients 80 years and older treated with transcatheter aortic valve implantation (TAVI) or surgical aortic valve replacement (SAVR)

Variables	Total (*N* = 143) Mean or count	± SD or (percent)	TAVI *n* = 65 Mean or count	± SD or (percent)	SAVR *n* = 78 Mean or count	± SD or (percent)	Univariate *p* value
Age (years)	83.5	± 2.7	84.8	± 2.8	82.4	± 2.0	< 0.001
Female	81	(57%)	41	(63%)	40	(51%)	0.16
Marital status							0.18
Married	77	(54%)	31	(48%)	46	(59%)	
Cohabitation status							0.13
Live alone	67	(47%)	35	(54%)	32	(41%)	
SOF—Frailty Index							0.11
Robust	48	(34%)	16	(25%)	32	(41%)	
Prefrail	39	(27%)	21	(32%)	18	(23%)	
Frail	56	(27%)	28	(43%)	28	(36%)	
MMSE	27.2	± 2.9	26.5	± 3.1	27.8	± 2.6	0.007
Barthel Index mean	18.9	± 1.5	18.8	± 1.5	19.0	± 1.5	0.37
BMI (kg/m^2^)	25.5	± 4.1	25.0	± 4.4	25.9	± 3.9	0.20
Charlson Comorbidity Index	2.1	± 1.2	2.5	± 1.3	1.8	± 1.0	< 0.001
Logistic EuroScore*	14.0	± 9.2	19.6	± 10.6	9.4	± 3.6	< 0.001
NYHA function class							< 0.001
I–II	48	(38%)	11	(20%)	37	(51%)	
III–IV	80	(62%)	45	(80%)	35	(49%)	
Left ventricle ejection fraction (%)	56.4	± 10.3	55.9	± 10.1	56.8	± 10.5	0.59
Max aorta gradient (mmHg)	79.3	± 24.9	74.4	± 23.8	83.6	± 25.2	0.03
Mean aorta gradient (mmHg)	48.2	± 16.6	45.6	± 16.3	50.6	± 16.7	0.08
Aortic valve area (cm^2^/m^2^)	0.4	± 0.2	0.4	± 0.1	0.4	± 0.2	0.64
ASA classification							< 0.001
III	120	(84%)	44	(68%)	76	(97%)	
IV	23	(16%)	21	(32%)	2	(3%)	

*ASA* American Society of Anesthesiologists Classification, *BMI* Body Mass Index, *NYHA Function Class* New York Heart Association Function Classification, *MMSE* Mini Mental Status Examination, *SOF* Study of Osteoporotic Fractures

*p value based on log-transformed values

**Fig. 1:**
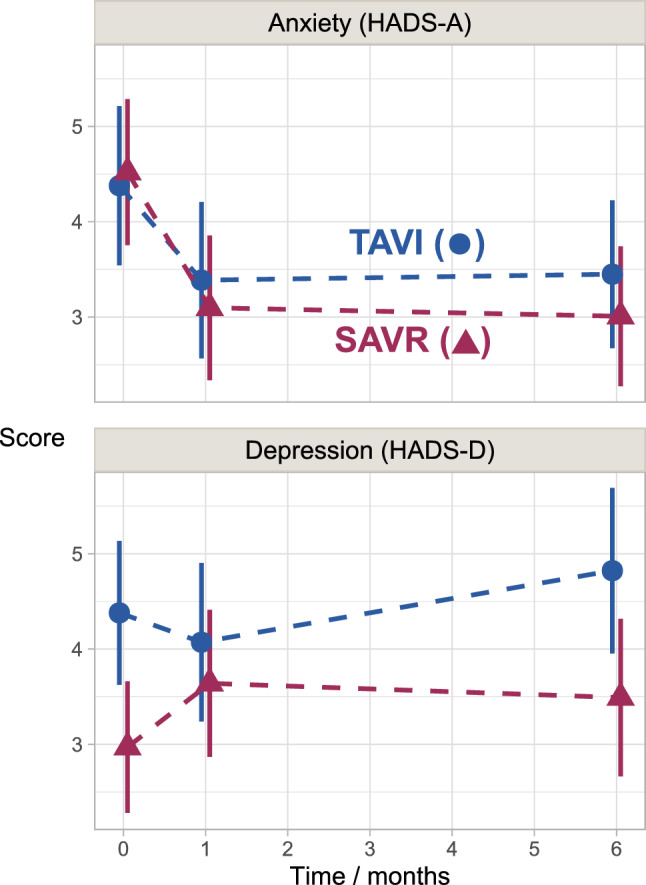
Estimated mean scores for anxiety (HADS-A) and depression (HADS-D) (721 measurements) in patients aged 80 years and older (N = 142 patients) following surgery aortic valve replacement (SAVR, 78 patients, 385 measurements) or transcatheter aortic valve implantation (TAVI, 64 patients, 336 measurements) Based on longitudinal models taking into account missing data

Estimated HADS mean scores for anxiety and depression are shown in Table 2 and Fig. 1. For anxiety (HADS-A), there was a clear change over time (*P* < 0.001), with a large reduction from baseline to one month, and stable mean scores from one to six months. This pattern was similar in both TAVI and SAVR patients. For depression (HADS-D), there was no evidence of change over time in either group (*P* = 0.21). Results according to a cut-off score ≥ 8 for anxiety and depression in both treatment groups are as follows:

**Table 2:** Estimated mean scores for anxiety (HADS-A) and depression (HADS-D), in patients aged 80 years and older (N = 142 patients) following surgery aortic valve replacement (SAVR, 78 patients, 385 measurements) or transcatheter aortic valve implantation (TAVI, 64 patients, 336 measurements)

Subscale	Time	SAVR	TAVI	Diff	95% CI			*p* value
HADS-A	Baseline	4.5	4.4	− 0.1	− 1.3	to	1.0	0.81
	One month	3.1	3.4	0.3	− 0.8	to	1.4	0.61
	Six months	3.0	3.4	0.4	− 0.6	to	1.5	0.42
HADS-D	Baseline	3.0	4.4	1.4	0.4	to	2.4	0.008
	One month	3.6	4.1	0.4	− 0.7	to	1.6	0.46
	Six months	3.5	4.8	1.3	0.1	to	2.5	0.03

Based on longitudinal models taking into account missing data

*SAVR* surgical aortic valve replacement, *TAVI* transcatheter aortic valve implantation, *Diff.* difference, *CI* confidence interval, *HADS-A* Hospital anxiety and depression scale, anxiety subscale, *HADS-D* Hospital anxiety and depression scale, depression subscale

### HADS-A TAVI Group

At baseline, 11% (7/62) of the TAVI patients had scores indicating possible anxiety. At one and six months after the procedure, the corresponding percentages were 8% (4/53) and 9% (5/53).

### HADS-A SAVR Group

At baseline, 18% (13/73) of the SAVR patients had scores indicating possible anxiety. At one and six months after the procedure, the corresponding percentages were 11% (7/62) and 9% (5/57).

### HADS-D TAVI Group

At baseline, 15% (9/62) of the TAVI patients had scores indicating possible depression. At one and six months after the procedure, the corresponding percentages were 11% (6/53) and 17% (9/53).

### HADS-D SAVR Group

At baseline, 11% (8/74) of the SAVR patients had scores indicating possible depression. At one and six months after the procedure, the corresponding percentages were 15% (9/62) and 12% (7/57).

## Discussion

In this study of patients aged 80 years and older receiving elective cardiac procedure for AS with TAVI or SAVR, we were able to identify a clear reduction in anxiety scores between the time of inclusion and follow-up. These changes remained stable between the one- and six-month time points for both treatment modalities. For depression, there was no evidence of change over time in either treatment group. However, the confidence intervals for the mean scores were wide and thus consistent with some (minor) change over time. Overall, TAVI patients were older, had lower ADL and cognitive function and a higher logistic EuroSCORE, were often placed in more severe ASA categories, and had higher mean scores on depression than SAVR patients.

Preoperative anxiety is not uncommon among patients undergoing elective surgery [35] and especially before cardiac surgery [36]. It is not rare to have concerns regarding the outcome and possible complications of an invasive procedure intended to treat a life-threatening condition such as AS. However, preoperative anxiety is a risk factor for negative outcomes that cannot be underestimated as it has been linked to lower quality of life and cognitive performance, a greater need for information, poorer memory and attention, longer hospitalization, depressive symptoms, and increased physical disability [37]. Considering the growing numbers of elderly patients in need of advanced treatment for AS, it would be sensible to identify particularly anxious patients preoperatively and to implement measures to prevent or reduce the effect of the condition.

Increased age has been associated with higher levels of anxiety [2] and it has been suggested that the condition might lead to higher risk for major adverse cardiovascular events and poor patient-reported outcomes [4]. Yet, with some few exceptions, anxiety in the oldest group of patients, those 80 years-old and older, has been understudied [14]. Our findings show that, despite several significant differences before treatment, the mean anxiety scores in both the TAVI and SAVR groups were similar at baseline (Table 2 and Fig. 1). Anxiety was also measured in Chinese patients aged 70 years and older (mean age 77.6 ± 4.6) scheduled for advanced treatment of AS [38]. These patients reported higher anxiety scores (6.9 ± 2.6 for TAVI and 7.5 ± 2.5 for SAVR) [38] than those included in our study. Higher mean scores for anxiety (9.9 ± 1.9) were also present in a German study of TAVI patients, although its population was slightly younger (mean age 77.8 ± 7.7 years) [39].

While HADS-A mean scores in our group of patients showed a substantial decline one and six months after treatment, mean scores in the Chinese group increased at the one-month follow-up (10.9 ± 3.8 for TAVI and 12.2 ± 3.4 for SAVR) and remained high for eight months after treatment (10.3 ± 3.7 for TAVI and 12.1 ± 2.8 for SAVR) [38]. In the German study, anxiety scores with TAVI declined from 9.9 ± 1.9 at baseline to 7.3 ± 3.5 six weeks after treatment [39]. Differences in HADS-A scores at follow-up among the patients in our study and the German study compared to those in the Chinese study might be linked to socio-geographical differences. Anxiety and/or depression following TAVI and SAVR was retrospectively examined in a recent study [40]. A higher rate of incident anxiety and/or depression following SAVR (when compared to TAVI) was found, yet the risk for these two conditions was higher in patients experiencing complications regardless of treatment type [40]. While the mean age for patients treated with TAVI in the mentioned study was high (80.8 years, SD ± 8.6), the mean age for those receiving SAVR was much lower (65.8 years, SD ± 9.0) than in our study group. Besides, anxiety and/or depression was not measured before treatment [40].

The TAVI and SAVR groups in our study reported significantly different levels of depression at baseline (Table 2 and Fig. 1). In general, our patients reported lower scores for depression compared to other studies. For instance, baseline mean scores of HADS-D in the Chinese study were 7.1 ± 3.1 in TAVI patients and 7.9 ± 2.9 in SAVR patients. This represents a difference of 3 points above the mean scores of both TAVI and SAVR patients in our study (Table 2). Bäz et al. [39] reported baseline mean scores in HADS-D that were 6 points higher (10.8 ± 2.8) than those of the TAVI patients in our group.

Most of the patients in our study reported lower mean scores for anxiety and depression compared to previous studies with similar populations and at comparable data collection times [38, 39]. The results from our study might indicate that patients with mental health problems were less likely to be referred or selected for cardiac intervention. On the other hand, this may also represent the prevalence (and demography) of anxiety and depression in Northern Europe, which peaks around middle age and decreases after retirement, as reported in the 2022 *World Mental Health Report* [2]. Further support of this phenomenon can be found in two large longitudinal population studies that identified a reduction in the prevalence of anxiety in the elderly [41, 42]. Yet, we do believe that there are possibilities to reduce mental stress at baseline and postoperatively. Information regarding the relatively low mortality and morbidity risk of cardiac intervention for AS may reduce patients’ anxiety before undergoing the procedure. Additionally, information about the potential physical improvement and better quality of life following surgery may also be beneficial to reduce postoperative anxiety and depression. Such informative intervention is an important message to all health personnel during all phases of treatment.

Among the strengths of this study is its prospective design, the use of valid and reliable measurement tools, and the inclusion of patients 80 years and older scheduled for advanced cardiac treatment with SAVR or TAVI. The centralized cardiac treatment at a tertiary university hospital for this part of the country ensured inclusion of a representative group of patients 80 years of age and older. Besides, only 2% of eligible patients refused to participate. These factors argue therefore for generalizability.

A limitation of our study is that it was not designed as a randomized controlled trial. However, at the time data collection was performed, randomization was not possible because TAVI and SAVR were used to treat distinctly different target groups [12]. Another limitation is the modest size of the studied cohort. Yet, the tertiary university hospital that performed the procedures has the overall responsibility for the specialist healthcare service in western Norway, covering over a million residents. The potential impact of anxiety and depression measured exclusively in patients healthy enough to return to the hospital for testing could have provided additional information. This is an issue we intend to examine closer in future studies. Even though we present secondary analyses of existing data, few studies have focused on patients aged 80 years and older, and increased knowledge about older patients with cardiovascular disease is warranted [43].

In conclusion, in a population consisting of patients aged 80 years and older with severe AS, treatment with SAVR or TAVI was associated with a reduction in anxiety scores between the time of inclusion and the follow-up times. For depression, there was no evidence of change over time in either treatment group. Our results highlight the importance of screening for mental health issues such as anxiety and depression, of longitudinal monitoring and follow-up of these conditions, and of potentially referral and treatment in the oldest group of patients.

### Supplementary Information

Below is the link to the electronic supplementary material.

Supplementary file1 (PDF 199 KB)

## Data Availability

Data availabily upon request.
