# Supplementary material for: Anxiety and depression in patients aged 80 years and older following aortic valve therapy. A six-month follow-up study
Source: Aging Clin Exp Res. 2023 Aug 30;35(11):2463–70. doi: 10.1007/s40520-023-02541-5 (PMC10628009; doi:10.1007/s40520-023-02541-5)

Anxiety and depression in patients 80 years and older following aortic valve therapy. A 6-month follow-up study

Aim: to evaluate changes in depression and anxiety from before intervention to follow-up at 1 and 6 months in patients 80 years and older and eligible for treatment with transcatheter aortic valve implantation (TAVI) or surgical aortic valve replacement (SAVR).

Prospective cohort study
tertiary university hospital

143 patients
≥ 80 years with
severe aortic stenosis

Anxiety and depression measured with the Hospital Anxiety and Depression Scale (HADS) at baseline, 1- and 6- months after TAVI or SAVR

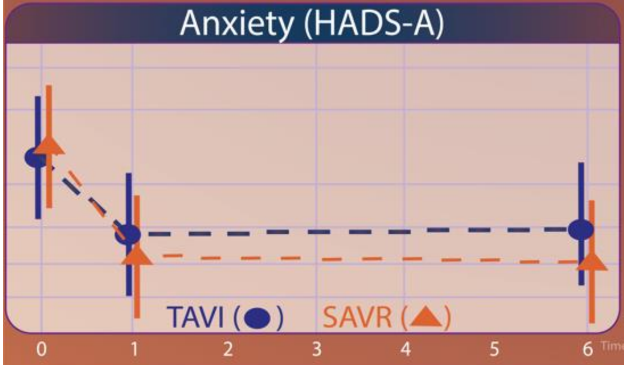

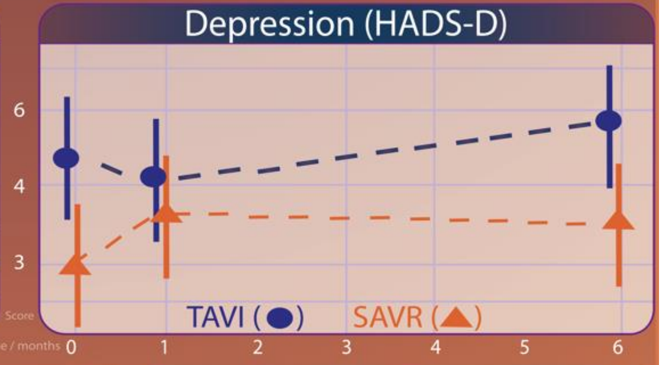

Treatment with SAVR or TAVI in patients ≥80 years of age was associated with reduction in anxiety scores between inclusion and follow-up times.
For depression, there was no significant difference over time in either treatment group.

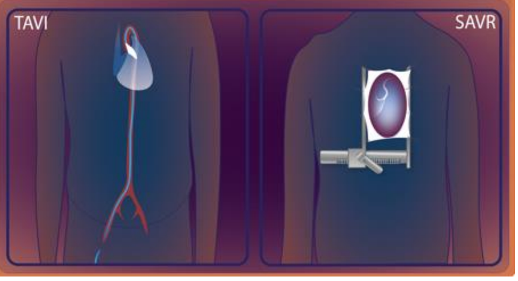

Supplement: Supplementary file 1 — Supplementary file1 (PDF 199 KB) [file 40520_2023_2541_MOESM1_ESM.pdf]
